# High CO_2_ Levels Impair Alveolar Epithelial Function Independently of pH

**DOI:** 10.1371/journal.pone.0001238

**Published:** 2007-11-28

**Authors:** Arturo Briva, István Vadász, Emilia Lecuona, Lynn C. Welch, Jiwang Chen, Laura A. Dada, Humberto E. Trejo, Vidas Dumasius, Zaher S. Azzam, Pavlos M. Myrianthefs, Daniel Batlle, Yosef Gruenbaum, Jacob I. Sznajder

**Affiliations:** 1 Division of Pulmonary and Critical Care Medicine, Feinberg School of Medicine, Northwestern University, Chicago, Illinois, United States of America; 2 Division of Nephrology, Feinberg School of Medicine, Northwestern University, Chicago, Illinois, United States of America; 3 Departamento de Fisiopatología, Facultad de Medicina, Universidad de la Republica, Montevideo, Uruguay; 4 University of Giessen Lung Center, Justus Liebig University, Giessen, Germany; 5 Ruth & Bruce Rappaport Faculty of Medicine and Research Institute, Technion-Israel Institute of Technology, Haifa, Israel; 6 Intensive Care Unit, Athens University, “KAT” General Hospital, Athens, Greece; 7 Department of Genetics, Hebrew University of Jerusalem, Jerusalem, Israel; Naval Research Laboratory, United States of America

## Abstract

**Background:**

In patients with acute respiratory failure, gas exchange is impaired due to the accumulation of fluid in the lung airspaces. This life-threatening syndrome is treated with mechanical ventilation, which is adjusted to maintain gas exchange, but can be associated with the accumulation of carbon dioxide in the lung. Carbon dioxide (CO_2_) is a by-product of cellular energy utilization and its elimination is affected via alveolar epithelial cells. Signaling pathways sensitive to changes in CO_2_ levels were described in plants and neuronal mammalian cells. However, it has not been fully elucidated whether non-neuronal cells sense and respond to CO_2_. The Na,K-ATPase consumes ∼40% of the cellular metabolism to maintain cell homeostasis. Our study examines the effects of increased pCO_2_ on the epithelial Na,K-ATPase a major contributor to alveolar fluid reabsorption which is a marker of alveolar epithelial function.

**Principal Findings:**

We found that short-term increases in pCO_2_ impaired alveolar fluid reabsorption in rats. Also, we provide evidence that non-excitable, alveolar epithelial cells sense and respond to high levels of CO_2_, independently of extracellular and intracellular pH, by inhibiting Na,K-ATPase function, via activation of PKCζ which phosphorylates the Na,K-ATPase, causing it to endocytose from the plasma membrane into intracellular pools.

**Conclusions:**

Our data suggest that alveolar epithelial cells, through which CO_2_ is eliminated in mammals, are highly sensitive to hypercapnia. Elevated CO_2_ levels impair alveolar epithelial function, independently of pH, which is relevant in patients with lung diseases and altered alveolar gas exchange.

## Introduction

Pulmonary edema occurs in patients with congestive heart failure and acute respiratory distress syndrome and often requires mechanical ventilation [1], [2]. It has been proposed that to prevent ventilator induced lung injury, patients should be ventilated with low tidal volumes which may result in hypercapnia [3], [4]. Some investigators have proposed that “permissive hypercapnia” could be beneficial in patients with lung injury [5], [6]. More recent studies have suggested that hypercapnia may have deleterious effects on the lungs; however, there has not been an attempt to define whether these effects were due to high pCO_2 _levels or the associated acidosis [7]–[10].

Average human respiration generates approximately 450 liters of carbon dioxide (CO_2_) per day [11], which, together with CO_2_ produced from other sources, is removed from the atmosphere by plants during photosynthesis. The notion of a sensor for CO_2_ has been proposed in plants and insects. In plants, the stomata of guard cells close when exposed to high CO_2_ concentrations via utilization of specific signaling pathways [12] while in *Drosophila* a CO_2_-sensitive receptor has been described in the olfactory neurons [13]. Recently, it has been reported that mice also can detect CO_2_ through the olfactory system involving carbonic anhydrase [14]. The effects of hypercapnia on excitable cells are well characterized and include depolarization of glomus cells, which trigger an increase in alveolar ventilation to maintain normal CO_2_ levels in the body [15]. In contrast, the effects of CO_2_ on non-excitable mammalian cells are not well understood. In vascular smooth muscle cells increased CO_2_ levels have been shown to activate mechanisms of cell adaptation, however, they were thought to be due to the changes in pH occurring during hypercapnia [16]. A recent report has suggested that renal epithelial cells respond to changes in CO_2_ concentrations via yet unidentified mechanisms [17].

Active Na^+^ transport effects edema clearance from the lungs via apically located sodium channels and basolateral Na,K-ATPase with water following iso-osmotically the Na^+^ gradient [18]–[20]. The Na,K-ATPase, a major modulator of cellular homeostasis, is expressed in all mammalian cells. It consists of a catalytic α-subunit and a regulatory β-subunit to exchange Na^+^ and K^+^ across the plasma membrane, consuming ∼40% of the energy of the cell in this process [21]. Inhibition of Na,K-ATPase activity can result from a decrease in the number of Na,K-ATPase molecules at the plasma membrane, usually via endocytosis and subsequent degradation of Na,K-ATPase proteins [22].

We have reported that hypercapnia decreases alveolar fluid reabsorption (AFR) in rats, however, carbonic anhydrase activity did not have an effect on AFR [23]. Here, we set out to determine whether the non-excitable alveolar epithelial cell, the site of CO_2_ elimination in mammals, is affected by elevated CO_2_ levels or the associated acidosis, focusing on the Na,K-ATPase and the alveolar epithelial function.

## Results

### High CO_2_ levels impair alveolar fluid reabsorption independently of pH

**Figure 1:**
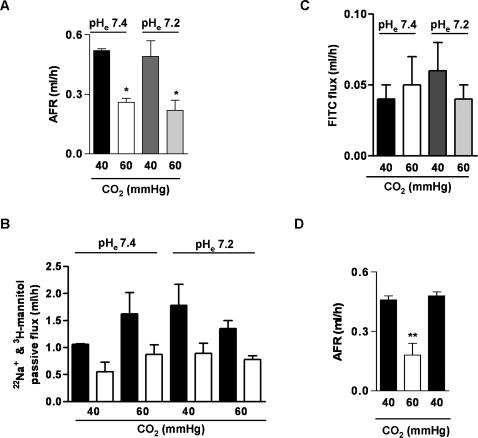
High CO_2_ levels impair alveolar epithelial function in rats . (A) Isolated rat lungs were perfused for 1 h with 40 or 60 mmHg CO_2 _with pH_e_: 7.4 or pH_e_: 7.2 and alveolar fluid reabsorbtion (AFR) was measured as described in the experimental procedures. Graph represents the mean±SEM, (n = 5). (B) Passive movement of Na^+^ (closed bars) and ^3^H-Mannitol (open bars) was measured as described in detail in the supplementary methods. Graph represents the mean±SEM (n = 5). Differences among groups were not statistically significant. (C) Albumin flux from the pulmonary circulation into the alveolar space was determined from the fraction of fluorescein isothiocyanate (FITC)-labeled albumin, placed in the perfusate that appeared in the alveolar instillate during the experimental protocol. Graph represents the mean±SEM, (n = 5). Differences among groups were not statistically significant. (D) A three hour experiment was performed in isolated rat lungs. Lungs were perfused for 1h with 40 mmHg CO_2_, switched to 60 mmHg CO_2_ for the second hour, and back to 40 mmHg CO_2_ for the third hour (pH_e_: 7.4). Alveolar fluid reabsorption (AFR) was measured as described in the experimental procedures. Graph represents the mean±SEM of 5 independent experiments. Bars in panels A, B and C represent the mean from different groups of animals. Bars in panel D represent the mean of single samples from the same group of animals. pH_e_: extracellular pH. *p<0.05; **p<0.01.

**Figure 2:**
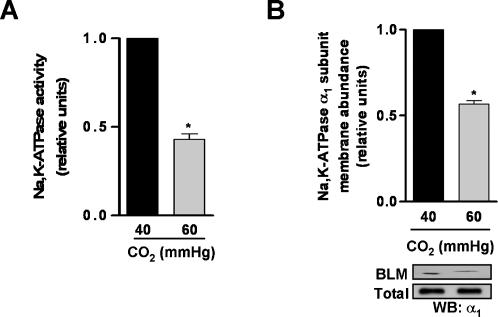
Na,K-ATPase function is impaired in rat lungs exposed to hypercapnic acidosis. (A) Basolateral membranes (BLM) were purified from the peripheral lung tissue of rat lungs exposed to 40 mmHg CO_2 _(pH_e_: 7.4) or 60 mmHg CO_2 _(pH_e_: 7.2), and Na,K-ATPase activity was measured as [γ-^32^P]ATP hydrolysis. Graph represents the mean±SEM, (n = 3). (B) BLM and total membranes were purified from the peripheral lung tissue of rat lungs treated as (A), and Na,K-ATPase protein abundance was assessed by Western blot. Graph represents the mean±SEM, (n = 3). Representative blots of Na,K-ATPase α_1_-subunit at the BLM and total membrane protein abundance are shown. pH_e_: extracellular pH. * p<0.05.

Alveolar fluid reabsorption (AFR), a major function of the lung epithelium, is contributed by apical Na^+^ channels and basolateral Na,K-ATPase [24], [25]. During lung injury, the alveolar epithelial Na,K-ATPase function is typically inhibited in association with impaired AFR [26]. As depicted in Figure 1A, high CO_2_ levels, independently of extracellular pH, decreased AFR by ∼50%, without affecting the passive fluxes of small or large solutes indicating that there were no changes in epithelial barrier permeability (Figure 1B and 1C). Also, the AFR inhibition by short-term hypercapnia was reversible within one hour of normalization of CO_2_ levels (Figure 1D). Na,K-ATPase activity and protein abundance was decreased at the basolateral membranes isolated from hypercapnia exposed peripheral lung tissue samples (Figure 2A and 2B).

### High CO_2_ levels regulate Na,K-ATPase independently of pH

**Figure 3:**
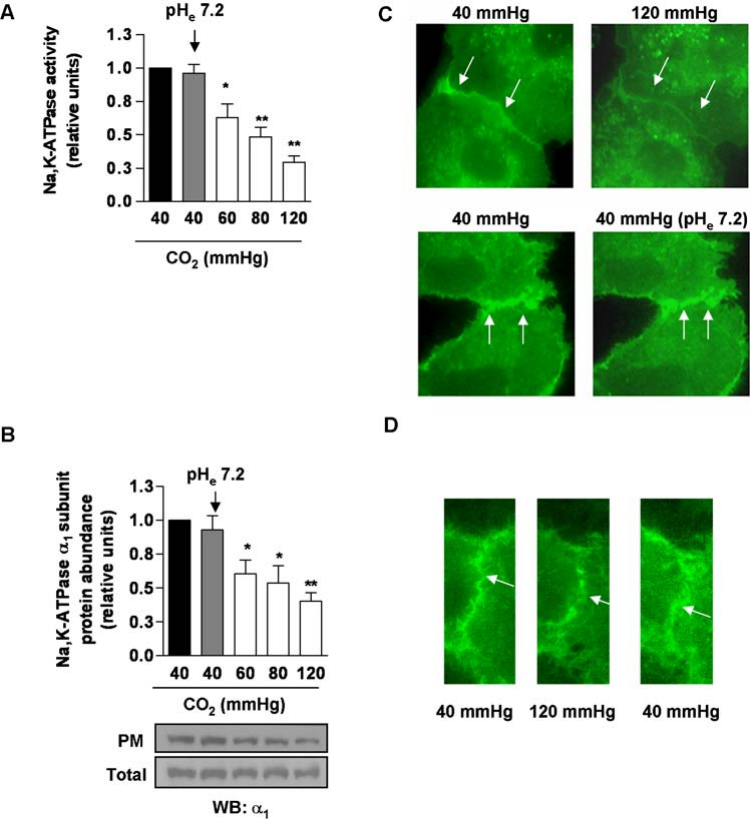
High CO_2 _levels impair Na, K-ATPase activity independently of pH. (A) ATII cells were exposed to 40, 60, 80 and 120 mmHg CO_2 _with extracellular pH (pH_e_): 7.4 or to 40 mmHg CO_2 _with pH_e_: 7.2 for 30 min, and Na,K-ATPase activity was measured as [γ-^32^P]ATP hydrolysis. Graph represents the mean±SEM, (n = 5). (B) ATII cells were treated as described in (A) and the Na,K-ATPase protein abundance at the plasma membrane (PM) was determined by biotin-streptavidin pull down and subsequent Western blot. Graph represents the mean±SEM, (n = 5). Representative blots of Na,K-ATPase α_1_-subunit at the PM and total protein abundance are shown. (C) Live cell imaging of GFPα_1_-A549. Cells were exposed to 40 mmHg CO_2 _(pH_e_: 7.4) for 10 min (left panels) and then switched to 120 mmHg CO_2 _(pH_e_: 7.4) (right, upper panel) or to 40 mmHg CO_2 _(pH_e_: 7.2) (right, lower panel) for 30 min. White arrows indicate the plasma membrane Na,K-ATPase. (D) Live cell imaging of GFPα_1_-A549. Cells were exposed to 40 mmHg CO_2 _(pH_e_: 7.4) for 10 min (left panel), switched to 120 mmHg CO_2 _(pH_e_: 7.4) (middle panel) for 30 min and switched back to 40 mmHg CO_2 _(pH_e_: 7.4) for 60 min (left panel). White arrows indicate the plasma membrane Na,K-ATPase * p<0.05, ** p<0.01.

**Figure 4:**
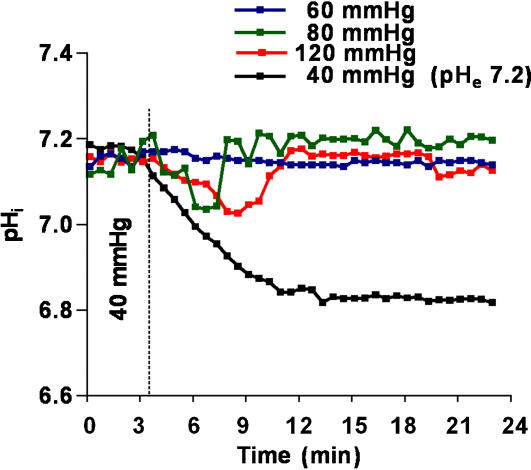
Effects of high CO_2_ and extracellular acidosis on intracellular pH. Intracelluar pH (pH_i_) was measured in real time as the change in fluorescence intensity of ATII cells loaded with BCECF/AM and exposed approximately 3 min to 40 mmHg, and then for the indicated time to 60, 80 and 120 mmHg CO_2 _with pH_e_: 7.4, or 40 mmHg with pH_e_: 7.2. pH_e_: extracellular pH.

We investigated whether cultured alveolar epithelial cells were sensitive to elevated levels of CO_2_ by examining the effects of CO_2_ on Na,K-ATPase function. In rat alveolar epithelial type II (ATII) cells exposed to increasing levels of CO_2_ (while buffering the medium to a pH of 7.40) for 30 minutes, the Na,K-ATPase catalytic activity decreased in a concentration-dependent manner (Figure 3A) due to the Na,K-ATPase α_1_-subunit endocytosis from the plasma membrane into intracellular compartments, as assessed by cell surface biotinylation (Figure 3B) and live cell imaging in GFP α_1_-A549 (Figure 3C). Also, the Na,K-ATPase α_1_-subunit endocytosis induced by short-term hypercapnia was reversible within one hour of normalization of CO_2_ levels (Figure 3D). These effects were due to high CO_2_ levels and not to alterations in extracellular pH (pH_e_), because neither the Na,K-ATPase activity nor the Na,K-ATPase protein abundance at the plasma membrane were affected by low pH_e_ levels at constant CO_2_ concentrations (Figure 3A, 3B and 3C). We also observed that the intracellular pH (pH_i_) markedly decreased when cells were incubated at a pH_e_ of 7.20 and normal CO_2_ concentration (40 mmHg) (Figure 4). In contrast, incubating cells with increasing levels of CO_2_ at pH_e_ of 7.40 resulted in a mild and transient decrease in pH_i_ which rapidly returned to baseline levels. These data suggest that high CO_2_ levels in alveolar epithelial cells, independently of pH, promote the endocytosis of Na,K-ATPase and thus inhibit its activity.

### CO_2_ activates PKCζ which phosphorylates Na,K-ATPase α_1_-subunit at Ser-18

**Figure 5:**
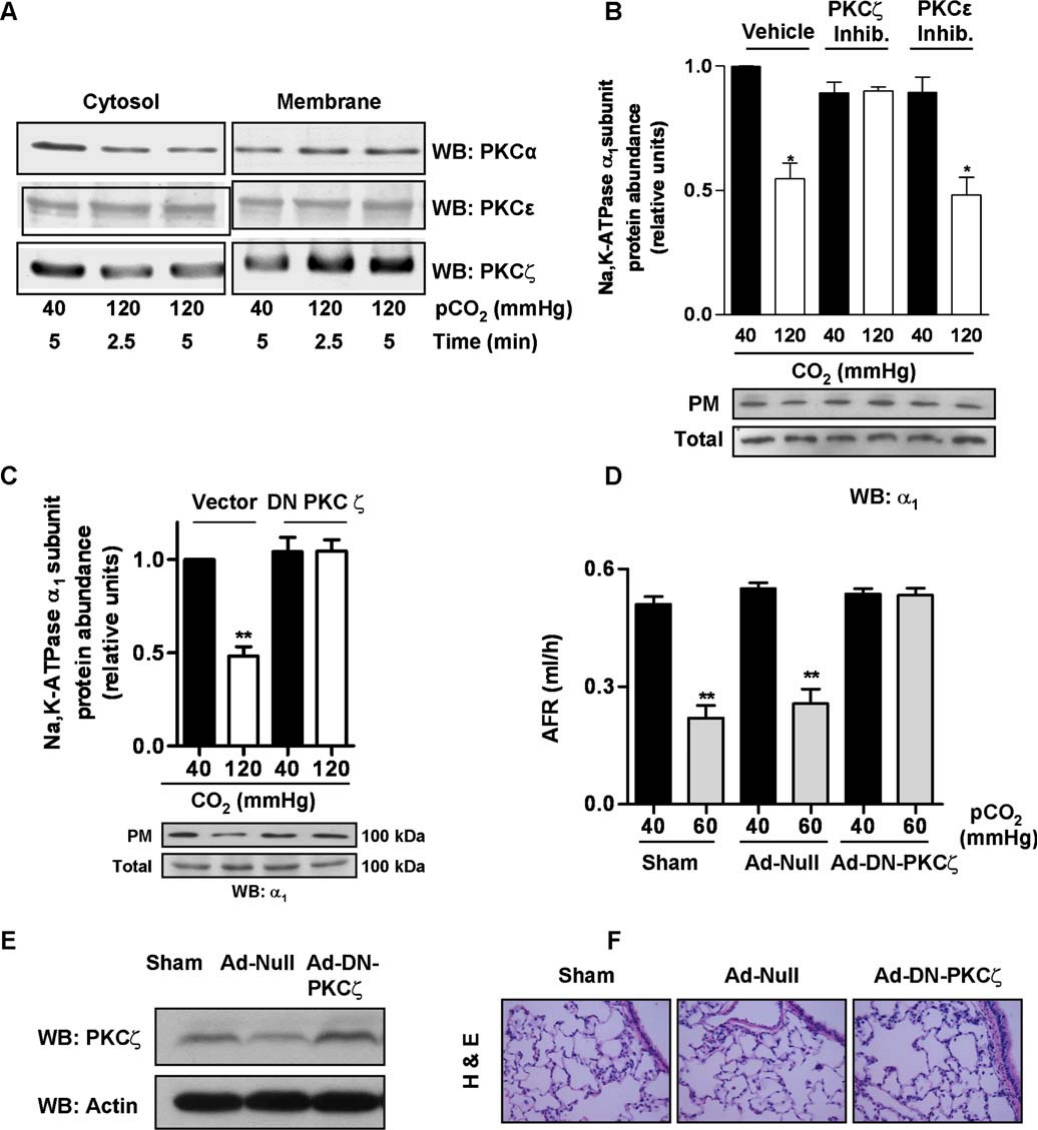
Role of PKCζ in CO_2_-induced Na,K-ATPase α_1_-subunit endocytosis. (A) ATII cells were exposed for the indicated times to 40 or 120 mmHg CO_2 _(pH_e_: 7.4), cytosolic and 1% Triton X-100 soluble fractions were isolated, and translocation of different PKC isoforms was determined by Western blot with specific antibodies. Representative blots for PKCα, PKCε and PKCζ are shown (n = 3). (B) ATII cells were incubated with vehicle, 5 µM PKCε inhibitory peptide or 0.1 µM PKCζ inhibitory peptide 1 h prior to being exposed to 40 or 120 mmHg CO_2 _(pH_e_: 7.4) for 30 min. Na,K-ATPase protein abundance at the PM was determined by biotin-streptavidin pull down and subsequent Western blot. Graph represents the mean±SEM, (n = 5). Representative blots of Na,K-ATPase α_1_-subunit at the PM and total protein abundance are shown. (C) A549 cells expressing an empty vector or a DN-PKCζ were exposed to 40 or 120 mmHg CO_2 _(pH_e_: 7.4) for 30 min. The Na,K-ATPase protein abundance at the PM was determined as above. Graph represents the mean±SEM, (n = 5). Representative blots of Na,K-ATPase α_1_-subunit at the PM and total protein abundance are shown. (D) Isolated rat lungs from rats infected with Sham-surfactant, with null adenoviral vector (Ad-null), and adenoviral vector with DN PKCζ construct (Ad-DN-PKCζ) were perfused for 1 h with 40 mmHg CO_2 _(pH_e_: 7.4) or with 60 mmHg CO_2 _(pH_e_: 7.2), and AFR was measured as described in the Methods section. Graph represents the mean±SEM, (n = 5). (E) Lungs from rats infected with Sham, Ad-null and Ad-DN-PKCζ were thoroughly rinsed with ice-cold PBS, tissue was homogenized, and the abundance of PKCζ protein abundance was determined by Western blot. Representative Western blots of PKCζ and actin (loading control) are shown. (F) Lung tissues from rats infected with Sham, Ad-null and Ad-DN-PKCζ were thoroughly rinsed with ice-cold PBS and fixed in 4% paraformaldehyde. Hematoxylin and eosin (H&E) staining was performed as described in the Online Data Supplement. Magnification x40. *p<0.05, **p<0.01.

Plasma membrane proteins undergo post-translational modifications, such as phosphorylation or oxidation which leads them to endocytose [27]. Previous reports have suggested that upon stimulation, members of the PKC family translocate from the cytosol to the membrane compartments and phosphorylate the plasma membrane Na,K-ATPase leading to its endocytosis. Specifically, PKCζ has been shown to directly phosphorylate the Na,K-ATPase α_1_-subunit at Ser-18 [28]. Here, we observed that exposing cells to high CO_2_ levels translocated both classical (α) and atypical (ζ) but not novel (ε) PKC isotypes to the plasma membrane (Figure 5A). To determine whether PKCζ was involved in CO_2_-induced Na,K-ATPase endocytosis, we incubated the cells with a selective PKCζ inhibitory peptide (1 h, 0.1 µM) and found that in cells incubated with this inhibitory peptide, but not with a selective PKCε inhibitory peptide (1 h, 5 µM), CO_2_-induced Na,K-ATPase endocytosis was prevented (Figure 5B). To further determine the role of PKCζ, we exposed cells expressing a dominant negative PKCζ, (DN PKCζ) to high levels of CO_2_ and compared them with cells expressing an empty vector. As shown in Figure 5C, high CO_2 _levels did not cause Na,K-ATPase endocytosis in cells expressing the DN PKCζ . To test whether these observations have physiological relevance, rats where infected with adenovirus coding for a DN PKCζ and as shown in Figure 5D, in the rats expressing the DN PKCζ the CO_2_-induced inhibition of the alveolar fluid reasorption was prevented.

**Figure 6:**
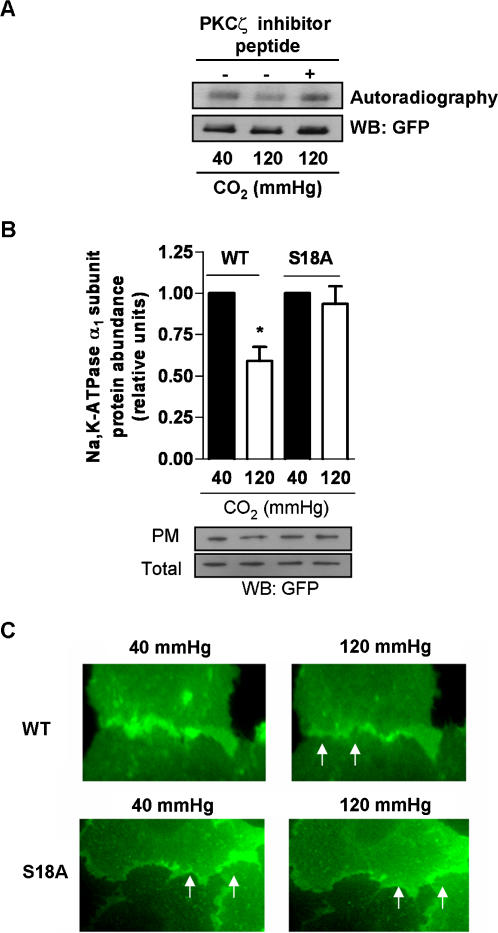
PKCζ phosphorylates the Na,K-ATPase α_1_-subunit at Ser 18. (A) *In vitro* “back phosphorylation” assay was performed (as described in the methods section) on the immunoprecipitated Na,K-ATPase α_1_-subunit from GFPα1-A549 cells exposed to 40 or 120 mmHg CO_2 _(pH_e_: 7.4) for 10 min in the presence or absence of PKCζ inhibitory peptide (0.1 µM, 1 h). Upper panel shows a representative autoradiography. Lower panel depicts a representative Western blot (n = 3; p<0.05 when comparing 40 mmHg vs 120 mmHg). (B) A549 cells expressing the rat GFPα_1_-subunit Na,K-ATPase (WT) or the rat GFP-S18A α_1_-subunit (S18A) were exposed to 40 or 120 mmHg CO_2 _(pH_e_: 7.4) for 30 min. The Na,K-ATPase protein abundance at the plasma membrane (PM) was determined by biotin-streptavidin pull down and subsequent Western blot. Graph represents the mean±SEM, (n = 5). Representative blots of Na,K-ATPase α_1_-subunit at the PM and total protein abundance are shown. (C) Live cell imaging of A549 cells expressing the rat GFPα_1_-subunit Na,K-ATPase (WT) or the rat GFP-S18Aα_1_-subunit (S18A). Cells were exposed to 40 mmHg CO_2 _(pH_e_: 7.4) for 10 min (left panels) and then switched to 120 mmHg CO_2 _(pH_e_: 7.4) for 30 min (right panels). White arrows indicate the plasma membrane Na,K-ATPase. * p<0.05.

High levels of CO_2_ induced Na,K-ATPase α_1_-subunit phosphorylation as assessed by a “back phosphorylation” assay (Figure 6A). This phosphorylation was prevented in cells treated with a PKCζ inhibitory peptide suggesting that the Na,K-ATPase is a target of PKCζ (Figure 6A). Furthermore, in A549 cells expressing a rat Na,K-ATPase α_1_-subunit where the Ser-18 residue was mutated to alanine (S18A), the high CO_2_-induced Na,K-ATPase endocytosis was prevented as assessed by cell surface biotinylation and live cell imaging with epi-fluorescent microscopy (Figure 6B and 6C).

## Discussion

Increased CO_2_ levels are observed in patients with impaired alveolar ventilation such as chronic obstructive pulmonary disease (COPD) and are predictive of poor prognosis [29]. In patients with lung injury on mechanical ventilation, acute hypercapnia is commonly a manifestation of hypoventilation and is associated with acidosis. Hypercapnic acidosis has been reported to impair cellular functions such as host inflammatory response and other deleterious effects including intracranial bleeding, decreased colonic Na^+^ transport and changes in pulmonary vascular resistance leading to ventilation/perfusion mismatch [30].

Our study was designed to determine whether short term high CO_2_ levels or the associated acidosis affect the alveolar epithelial function, assessed as active Na^+^ transport and thus alveolar fluid reabsorption. We observed that high CO_2_ levels impaired alveolar epithelial function which may be of importance for patients with lung injury, where inability to clear alveolar edema is associated with increased mortality [31]. To determine whether the impaired alveolar fluid reabsorption was due to high CO_2_ levels or to acidosis we bubbled into the pulmonary circulation high CO_2_ levels but buffered the solution to maintain a pH of 7.40 and also conducted experiments with normal CO_2_ levels but acidic pH. As shown in Figure 1A, high CO_2_ levels either with or without acidosis, resulted in impaired alveolar fluid reabsorption. These changes were reversible when normocapnia was restored (Figure 1D). Neither hypercapnia nor metabolic acidosis caused significant changes in epithelial permeability to small or large solutes.

We also studied the effects of CO_2_ on the alveolar epithelial cell Na,K-ATPase, a major contributor to alveolar fluid clearance. We incubated alveolar epithelial cells with high CO_2 _levels as compared to metabolic acidosis and observed that Na,K-ATPase activity and protein abundance at the plasma membrane decreased in high CO_2_ conditions at normal extracellular pH while lowering the pH with normal CO_2_ levels had no effect (see Figure 3). The changes in protein abundance at the plasma membrane occurred without affecting the total cell pools of the Na,K-ATPase consistent with the notion that the Na,K-ATPase were endocytosed from the plasma membrane into intracellular compartments and not degraded.

These data raise the question of whether these effects are due to a CO_2_ specific “sensor” in the alveolar epithelium or due to changes in intracellular pH. The notion of a sensor for high CO_2_ levels has been proposed in plants where stomata of Guard cells close when exposed to high CO_2_ levels [12]. Also, Zhou et al suggested that in renal cells, CO_2_ levels may be sensed outside the glomus cells in the carotid bodies [17]. Thus, we measured the intracellular pH in cells exposed to high CO_2_ levels at normal pH or normocapnia and low pH by modifying the extracellular perfusing solution and in both cases we observed a decrease in intracellular pH which normalized in the high CO_2_ exposed cells but remained low in cells exposed to low extracellular pH (Figure 4). Collectively, these data suggest that high CO_2_ levels and not acidosis triggered the endocytosis and thus the inhibition of Na,K-ATPase activity in alveolar epithelial cells.

Previous reports have suggested that inhibition of Na,K-ATPase activity is related to the phosphorylation status of the Na,K-ATPase via activation of different PKC isozymes [28], [32]. We found that PKCζ and PKCα isozymes were rapidly activated by high CO_2 _(Figure 5A), however, only PKCζ participated in the signaling pathway leading to Na,K-ATPase endocytosis and impaired alveolar fluid reabsorption. Furthermore, although we can not completely exclude the possibility that PKCζ may activate another kinase, our data strongly suggest that PKCζ phosphorylated the Na,K-ATPase α_1_- subunit as phosphorylation was prevented by a PKCζ inhibitory peptide (Figure 6A). It has been previously reported that Ser-18 in the Na,K-ATPase α_1_-subunit is the major site for PKC-mediated phosphorylation [33]. As shown in Figure 6, in rat GFP-S18A-α_1_-subunit mutant cells exposed to high CO_2_ levels the Na,K-ATPase protein abundance at the plasma membrane was unchanged suggesting that the phosphorylation of the Ser18 was necessary for the CO_2_-induced endocytosis.

In summary, we provide evidence that exposing alveolar epithelial cells to high CO_2_ levels affects the function of the alveolar epithelium, the primary site of CO_2_ elimination in mammals. Exposing cells to high concentrations of CO_2_, but not acidosis, resulted in activation of PKCζ which directly phosphorylated the Na,K-ATPase α_1_-subunit at the Ser-18 residue, triggering its endocytosis from the plasma membrane and causing a decrease in Na,K-ATPase function independently of pH changes. Notably, these effects were observed not only when cells and lungs were exposed to very high levels of CO_2_ (∼120 mmHg) but also at the clinically relevant levels in patients with respiratory failure or COPD (pCO_2_ ∼60–80 mmHg). Thus, we propose that changes in CO_2_ concentration are sensed not only by neuronal but also by non-excitable mammalian cells such as the alveolar epithelial cells. Identification of these sensing mechanisms and further elucidation of the CO_2_-mediated signaling pathways will not only further our understanding of a basic mechanism by which mammalian cells adapt to hypercapnia, but may also lead to novel therapeutic approaches in the treatment of lung diseases that are associated with poor alveolar ventilation.

## Materials and Methods

### Reagents

All cell culture reagents and G418 were from Mediatech Inc (Herndon, VA). Rat brain protein kinase C (PKC) was purchased from EMD Biosciences (San Diego, CA). Ouabain was from ICN Biomedicals Inc. (Aurora, OH).^ 22^Na^+ ^and [γ-^32^P] ATP were from GE Healthcare (Piscataway, NJ). Percoll was from Amersham Bioscience (Uppsala, Sweden). Myristoylated PKCζ peptide and PKCε v1-2 peptide were purchased from Biomol International (Plymouth Meeting, PA). ^3^H-mannitol was purchased from Perkin Elmer (Life Sciences, Inc, Boston, MA). All other chemicals were purchased from Sigma (St. Louis, MO). Na,K-ATPase α_1_ subunit monoclonal antibody (clone 464.6) was purchased from Upstate Biotechnology (Lake Placid, NY). Rabbit polyclonal PKCα (C-20), mouse monoclonal PKCε (E-5), mouse monoclonal PKCζ (H-1), mouse monoclonal GFP (B-2) antibodies, and Protein A/G plus were purchased from Santa Cruz (Santa Cruz, CA). Rabbit polyclonal anti-GFP antibody was from Clontech (Palo Alto, CA). Rabbit polyclonal anti-actin was from Sigma (St. Louis, MO). Secondary goat anti-mouse-HRP and goat anti-rabbit-HRP were from Bio-Rad (Hercules, CA).

### Animals

Pathogen-free male Sprague-Dawley rats weighing 320–350 g were used for the isolated lung model and male Sprague Dawley (200–225 g) were used for alveolar epithelial type II cell isolation (Harlan, Indianapolis, IN). All animals were provided food and water ad libitum, were maintained on a 12:12-h light-dark cycle, and were handled according to National Institutes of Health guidelines and to Institutional Animal Care and Use Committee approved experimental protocols.

### Isolated-perfused rat lung model

The isolated lung preparation has been described in detail previously [18]. Briefly, the lungs and heart of anesthetized rats were removed en bloc. The pulmonary artery and left atrium were catheterized and perfused continuously with a solution of 3% bovine serum albumin (BSA) in buffered physiological salt solution (135.5 mM Na^+^, 119.1 Cl^−^, 25 mM HCO_3_^−^, 4.1 mM K^+^, 2.8 mM Mg^+^, 2.5 mM Ca^+2^, 0.8 mM SO_4_^−2^, 8.3 mM glucose). Trace amounts of FITC-albumin was also added to the perfusate. The recirculating volume of the constant pressure perfusion system was 90 ml; arterial and venous pressures were set at 12 and 0 cm H_2_O respectively. The vascular pressures were recorded every 10 seconds with a multichannel recorder (Cyber Sense Inc. Nicholasville, KY). The lungs were immersed in a “pleural” bath (100 ml) filled with the same BSA solution. The entire system was maintained at 37°C in a water bath. Perfusate pH was maintained at 7.40 by bubbling with a gas mixture of 95%O_2_/5%CO_2_. The lungs were then instilled via the tracheal cannula in two sequential phases with a total of 5 ml volume of the BSA solution containing 0.1mg/ml Evans Blue Dye (EBD)-albumin, 0.02 µCi/ml of ^22^Na^+^ and 0.12 µCi/ml of ^3^H-mannitol. Samples were taken from the instillate, perfusate, and bath solutions after an equilibration time of 10 minutes from the instillation and again 60 minutes later. To ensure a homogenous sampling of the instillate, a volume of 2 ml was aspirated and reintroduced into the airspaces three times before removing each sample. All samples were centrifuged at 3000 g for 10 minutes. Absorbance analysis of the supernatant or EBD-albumin was performed at 620 nm in a Hitachi model U2000 spectrometer (Hitachi, San Jose, CA). Analysis of FITC-albumin (excitation 487 nm and emission 520 nm) was performed in a Perkin-Elmer fluorometer (model LS-3B, Perkin-Elmer, Oakbrook, IL).

The amount of instilled Evans blue dye albumin remains constant during the experimental protocol, so any change in its concentration at a given time reflects changes in the airspace volume. Differences in concentration of Evans blue dye albumin among samples taken from the instillate at the beginning and after a determined time reflect the amount of fluid that has been reabsorbed. The fraction of fluorescein isothiocyanate albumin that appears in the alveolar space during the experimental protocol was used to calculate the albumin flux from the pulmonary circulation into the alveolar space [18], [34].

Scintillation counts for ^22^Na^+^ and ^3^H-mannitol were measured in a Beckman beta counter (model LS 6500, Beckman Instruments Inc., Fullerton, CA). Levels of pH, pO_2_ and pCO_2_ were monitored and controlled to the experimentally set parameters by bubbling more or less CO_2_ and/or adding NaOH or HCl in the pulmonary circulation perfusate. Samples were quickly processed to avoid accidental degassing during measurement maneuvers.

The sodium concentration is equal and constant in all the compartments and since ^22^Na^+ ^is instilled only in the airspace, the disappearance of the radioactive tracer from the airspaces reflects the total or unidirectional Na^+^ outflux from the airspace (*J*_Na,out_) [18], [34]. The passive or bidirectional Na^+^ flux between the airspace and the other compartments is the difference between the unidirectional (*J*_Na,out_) and active Na^+ ^outflux (*J*_Na,,net_ = [Na^+^]*J*). The passive sodium movement can be calculated by:
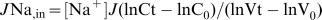
Where, C_0 _and Ct are the concentrations of ^22^Na^+^ initially and at time t respectively, and [Na^+^] is the constant sodium concentration in the buffered salt albumin solution. Similarly the mannitol flux (typically expressing the surface area permeability (PA) is given by:
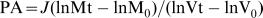



### Basolateral plasma membranes (BLM) isolation

BLM fractions were obtained from tissue collected from the distal 2–3 mm of rat right lungs following serial bronchoalveolar lavage (PBS 7 ml×5) and perfusion of the pulmonary artery (PBS×20 ml) as previously described [35]. Protein fractions enriched for the BLM domain were obtained generating a 16% percoll gradient [36].

### Determination of Na,K-ATPase activity

Na,K-ATPase activity was measured as previously described [22], [37]. Briefly, Na,K-ATPase activity was calculated from BLM from tissue or ATII cells exposed to the desired conditions, as the liberated ^32^P difference between the test samples (total ATPase activity) and samples assayed in reaction buffer with 2.5 mM ouabain but devoid of Na^+^ and K^+^ (nonspecific ATPase activity). Results are expressed as nmol of P_i_/mg of protein/hour.

### Total membranes from tissue

Tissue collected from the distal 2–3 mm of rat right lungs as described above was homogenized in homogenization buffer (1 mM EDTA, 1 mM EGTA, 10 mM Tris-HCl, pH: 7.5, 1 µg/ml leupeptin, 100 µg/ml TPCK and 1 mM PMSF), centrifuged at 500 g to discard nuclei and debris, and the supernatant was centrifuged at 100,000 g, 1 h, 4°C (TL ultracentrifuge, Beckman, Rotor TLA 100.2). Pellet was considered as the total membrane fraction.

### Western blot analysis

Protein concentration was quantified by Bradford assay [38] (Bio-Rad, Hercules, CA) and resolved in 10%–15% polyacrylamide gels (SDS-PAGE). Thereafter, proteins were transferred onto nitrocellulose membranes (Optitran, Schleider & Schuell, Keene, NH) using a semi-dry transfer apparatus (Bio-Rad, Hercules, CA). Incubation with specific antibodies was performed overnight at 4°C. Blots were developed with a chemiluminescence detection kit (PerkinElmer Life Sciences, Boston, MA) used as recommended by the manufacturer. The bands were quantified by densitometric scan (Image J 1.29X, National Institutes of Health)

### Alveolar epithelial type II cells isolation and cell culture

ATII cells were isolated as previously described [39]. Briefly, the lungs were perfused via the pulmonary artery, lavaged, and digested with elastase (3 U/ml; Worthington Biochemical, Freehold, NJ). ATII cells were purified by differential adherence to IgG-pretreated dishes, and cell viability was assessed by trypan blue exclusion (>95%). Cells were resuspended in Dulbecco's modified Eagle's medium containing 10% fetal bovine serum with 2 mM glutamine, 100 U/ml penicillin, 0.25 µg/ml amphotericin B, and 100 µg/ml streptomycin. Cells were incubated in a humidified atmosphere of 5% CO_2_-95% air at 37°C. The day of isolation and plating is designated cultured *day 0*. All experimental conditions were tested in *day 2* cells.

Human A549 cells (ATCC CCL 185) expressing the GFP-rat-Na,K-ATPase-α_1_-subunit (GFPα_1_-A549) [28], and GFP-S18A-rat-Na,K-ATPase-α_1_-subunit (40 121) were grown in Dulbecco's modified Eagle's medium (DMEM) supplemented with 10% fetal bovine serum, 100 U/ml penicillin, 100 µg/ml streptomycin and 3 µM ouabain to suppress the endogenous Na,K-ATPase α1 subunit. A549 cells expressing an empty vector and DN PKCζ were grown in the presence of G418 [28], [41]. Cells were incubated in a humidified atmosphere of 5% CO_2_/95% air at 37°C.

For the different experimental conditions, initial solutions were prepared with DMEM- Ham's F-12 medium-Tris base (3∶1∶0.5) containing 10% fetal bovine serum with 100 U/ml penicillin, and 100 µg/ml streptomycin. The buffer capacity of the media was modified by changing its initial pH with a Tris base in order to obtain a pH to 7.4 with the various CO_2_ levels (pCO_2_ of 40, 60, 80 and 120 mmHg). In some additional experiments, modeling extracellular acidosis, an initial pH of 6.8 was used to result in a final pH of 7.2 and a pCO_2_ of 40 mmHg. Then media was placed overnight in a humidified chamber (C-174 Chamber, Biospherix, Ltd., Redfield, NY) to achieve the desired CO_2_ and pH before starting the experimental protocols.

### Biotinylation of cell surface proteins

Cells were labeled for 20 minutes using 1 mg/ml EZ-link NHS-SS-biotin (Pierce Chemical Co., Rockford, IL). After labeling, the cells were rinsed three times with phosphate-buffered saline (PBS) containing 100 mM glycine to quench unreacted biotin and then lysed in modified radioimmunoprecipitation buffer (mRIPA; 50 mM Tris-HCl [pH 8], 150 mM NaCl, 1% NP-40, and 1% sodium deoxycholate, containing protease inhibitors-1 mM PMSF, 100ug TPCK, 10 µg/ml leupeptin [pH 7.4]). Aliquots (150 µg of protein) were incubated overnight at 4°C with end-over-end shaking in the presence of streptavidin beads (Pierce Chemical Co.). The beads were thoroughly washed and then resuspended in 30 µl of Laemmli sample buffer solution [42]. Proteins were analyzed by SDS-PAGE and Western blot.

### Live Cell Imaging

Epi-fluorescent microscopy images of GFPα_1_-A549 cells were obtained using a Nikon TE2000 (Nikon Instruments Inc, Melville NY) equipped with an environmental control system chamber (FCS2 system, Bioptechs Inc, Butler, PA) and a Planapo 60x 1.4 NA objective (Nikon Instruments Inc, Melville, NY). During imaging, the chamber was perfused with the specific culture media described above equilibrated to pCO_2_ of 40 or 120 mmHg and a pH_e_ of 7.4, while in other experiments a pCO_2_ was kept at 40 mmHg and pH_e_ was modified to 7.2. In experiments studying reversibility, cells were perfused for 10 min with media equilibrated to pCO_2_ of 40 mm Hg, changed to media equilibrated to pCO_2_ of 120 mm Hg for 30 min and back to media equilibrated to pCO_2_ of 40 mm Hg for 1 h. Under all experimental conditions oxygen was kept at 21%. Images were collected with a Cascade camera “TC285” EMCCD with on-chip multiplication gain (Photometrics; Tucson, AZ) driven by MetaMorph Software (Molecular Devices Corp. Downingtown, PA). For all the experiments exposure time was 0.5 seconds and to decrease phototoxic effects 0.25 neutral filter was used.

### Intracellular pH measurement

ATII were plated in circular glass cover slips, placed in a chamber for environmental control system for live cell imaging (FCS2 system, Bioptechs Inc, Butler, PA) and measurements were obtained with a multi-mode inverted Microscopy (Nikon TE2000, Nikon Instruments Inc, Melville NY). Cells were loaded with 1.5 µM 2′7′-bis-(carboxyethyl)-5,6-carboxyfluorescein (BCECF/AM) (Invitrogen, Carlsbad, CA) for 30 min at 37° C as described previously [43]. After dye loading, cover slips were placed in the chamber, maintained at 37° C and continuously perfused with equilibrated media. BCECF fluorescence in the chamber was monitored continuously through the desired excitation wavelength (500 and 440 nm) with an emission wavelength of 520 nm. For all the experiments the exposure time was 2 seconds. No neutral filter was used.

Signals were processed by MetaFluor Software (Molecular Devices Corp. Downingtown, PA).

### Cell fractionation

Cells were exposed to 120 mmHg of CO_2_ at 37°C for the desired times, placed on ice and washed twice with ice-cold PBS. Cells were scraped in homogenization buffer (1 mM EDTA, 1 mM EGTA, 10 mM Tris-HCl, pH: 7.5, 1 µg/ml leupeptin, 100 µg/ml TPCK and 1 mM PMSF) and homogenized by using a Dounce homogenizer. Homogenates were centrifuged at 500 g to discard nuclei and debris, and the supernatant was centrifuged at 100,000 g, 1 h, 4°C (TL ultracentrifuge, Beckman, Rotor TLA 100.2). The supernatant was considered the cytosolic fraction. The pellet containing the crude membrane fraction was resuspended in homogenization buffer +1% Triton X-100 and centrifuged at 100,000 g, 30 min, 4°C. The supernatant was considered the 1% Triton X-100 soluble fraction.

### Adenoviral infection

Rats were anesthetized with 40 mg/kg Nembutal intraperitoneally and intubated with a 14-gauge catheter prior to adenoviral infection. Three experimental groups were studied: Sham-surfactant (*n* = 5), null adenovirus (*n* = 5), and adenovirus expressing DN PKCζ (Cell Biolabs, San Diego, CA) (*n* = 5). A mixture of adenovirus (4×10^9^ PFU) in a 50% surfactant (Forest Laboratories Inc, St. Louis, MO), 50% dialysis buffer vehicle was administered in four aliquots of 250 µl. Immediately before instillation, a forced exhalation was achieved by circumferential compression of the thorax [44]. Compression was relinquished after endotracheal instillation of 250 µl of virus/vehicle followed by 500 µl of air. Infected animals were maintained in separate isolator cages for 7 days prior to conducting experimental protocols as described previously [44].

### Hematoxylin and eosin (HE) staining

Lung tissues were rinsed in ice-cold PBS and fixed in 4% paraformaldehyde overnight. Lungs were embedded in paraffin, and cut into 4 µm lung tissue sections, which were placed on glass slides. Slides were deparaffinized in xylene for 5 min (3 times) and then rehydrated in 100%, 95%, 70% ethanol and PBS. Hematoxylin and eosin (H&E) staining was performed. Briefly, slides were stained in hematoxylin for 3 min, rinsed in tap water, dipped in acid-alcohol 8–12 times, and finally rinsed in tap water. Next, slides were stained with eosin for 30 s and then dehydrated with 95% ethanol, 100% ethanol, and xylene. Images were observed with an Olympus Vanox-s equipped with an Olympus Japan 138132 objective and were captured using a Nikon Digital Camera System.

### Immunoprecipitation

GFPα_1_-A549 cells were incubated for 10 min at 5% and 20% CO_2_ in the presence or absence of the PKCζ inhibitory peptide. The incubation was terminated by placing the cells on ice, aspirating the media, washing twice with ice-cold PBS and adding immunoprecipitation buffer (20 mM Tris-HCl, 2 mM EGTA, 2 mM EDTA, 30 mM Na_4_P_2_O_7_, 30 mM NaF, 1 mM Na_3_VO_4_, 1 mM phenylmethylsulfonyl fluoride (PMSF), 100 µg/ml N-tosyl-L-phenylalanine chloromethyl ketone (TPCK), 10 µg/ml leupeptin [pH 7.4]). The cells were then scraped from the plates, frozen in liquid nitrogen, thawed, sonicated, frozen again, and centrifuged for 2 minutes at 14,000 *g*. After protein determination, SDS and Triton X-100 were added to each sample to a final concentration of 0.2% and 1%, respectively. Equal amounts of protein (700–1000 µg) were then incubated with polyclonal anti-GFP antibody for 2 hours at 4°C. Protein A/G PLUS-Agarose was added, and the samples were incubated overnight at 4°C. The samples were then washed twice with immunoprecipitation buffer supplemented with 0.2% SDS and 1% Triton X-100 and once with 20 mM Tris-HCl (pH 7.4).

### In vitro phosphorylation

The phosphorylation state of the immunoprecipitated Na,K-ATPase–GFPα_1_ subunit was assessed *in vitro* by the “*back phosphorylation*” method [28], [45]. The standard reaction mixture for *in vitro* phosphorylation of the Na,K-ATPase α_1_ subunit by purified PKC (150 ng per 150 µl, 30 minutes at 30°C) contained 10 mM MgCl_2_, 0.25 mM EGTA, 0.4 mM CaCl_2,_ 0.32 mg/ml L-α-phosphatidyl-L-serine, 0.03 mg/ml 1,2-dioleoyl-sn-glycerol (DAG), 0.1 mg/ml BSA, and 20 mM Tris-HCl (pH 7.5). The phosphorylation reaction was started by the addition of [γ-^32^P]ATP (final concentration, 100 µM; 1.3 µCi per sample). The reaction was stopped by placing the tubes on ice and washing the beads twice with 20 mM Tris-HCl (pH 7.4). Samples were analyzed by SDS-polyacrylamide gel, transferred to nitrocellulose membranes and autoradiographed.

### Data Analysis

Data are expressed as mean±SEM. Data were compared using ANOVA adjusted for multiple comparisons with the Dunnet test. When comparisons were performed between two groups of values significance was evaluated by non-paired Student's test and when comparison were made between repeated measures significance was evaluated by a paired Student́s test. A *p* value <0.05 was considered significant.
